# Optimization of fins arrangements for the square light emitting diode (LED) cooling through nanofluid-filled microchannel

**DOI:** 10.1038/s41598-021-91945-2

**Published:** 2021-06-15

**Authors:** Mohamed Bechir Ben Hamida, Mohammad Hatami

**Affiliations:** 1 College of Engineering, Department of Chemical Engineering, Ha’il University, Ha’il City 81481, Saudi Arabia; 2 Laboratory of Ionized Backgrounds and Reagents Studies (LEMIR), Preparatory Institute for Engineering Studies of Monastir (IPEIM), University of Monastir, Monastir City, Tunisia; 3 Mechanical Engineering Department, Ferdowsi University of Mashhad, Mashhad, Iran; 4 Higher School of Sciences and Technology of Hammam Sousse (ESSTHS), Department of Physics, University of Sousse, Sousse City, Tunisia

**Keywords:** Applied mathematics, Computational science, Computational nanotechnology

## Abstract

In current paper, a finned micro-channel is designed for the cooling application in Light Emitting Diode (LED), numerically using Galerkin weighted residual Finite Element Method (GFEM). Selected materials for LED-chip is GaN, Die from Si, Die-attach is made by Au-20Sn, substrate is copper and heat sink material is considered to be Al. To make a convection heat transfer for cooling process, Al_2_O_3_-water nanofluid is used as the cooling fluid flow through the micro-channel and tried to maximize the heat transfer efficiency by optimized geometry. For this aim, there geometry variables from the microchannel were selected and minimum possible geometry cases (11 cases) were proposed by Central composite design (CCD) and variables were optimized by the Response Surface Method (RSM). As a main result, parameter *B, i.e.* fin length had the most effect on the Nusselt number and Al_2_O_3_ nanoparticles with φ = 0.05 stated greatest heat transfer value. Also, different designs of fins arrangements, caused up to 6.5% increase in the nanofluid temperature which enhanced the LED cooling process.

## Introduction

Light Emitting Diode or LED lamps have taken the place of discharge lamps such as mercury discharge lamps^1,2^ and metal halide lamp^3,4^ due to better energy efficiency, small size, environmental friendliness, low UV radiation, easy control and low maintenance. So, due to this advantages of LED lamps, many researchers focused on its improvements and optimizations. For instance, Ben Salah and Ben Hamida^5,6^ investigated the heat transfer in LED geometries using air and PCM as alternative cooling materials. They also compared the results based on Ha and Graham^7^ study for a chip-on-board packing of LED arrays with high power. So, Ben Hamida et al.^8^ optimized some parameters such as: thickness, material and size of all subcomponents for single-chip LED to reduce the junction temperature and increase light output as well as device reliability. They considered six parts for the case modeling named die, die-attach, metallization, thermal interface material, substrate and heat sink to find the best material and concluded that GaN as LED-Chip material, SiC for Die material, Au as material metallization, 100In for Die-attach material, AIN DBC was the greatest substrate package, Copper for heat sink was the most suitable material and 100In solder as thermal Interface Material for designated parts. Also, other suitable materials are proposed by researchers such as CsPbBr3 as the perovskite light emitting diodes proposed by Jia et al.^9^. Yu et al.^10^ investigated the LED performance covered by cesium lead halide perovskite CsPbBr3 through a small organic molecule material named 1, 3, 5-tri (m-pyrid-3-yl-phenyl) benzene (TmPyPB) as a chemical stabilizer. In an experimental study about the LED cooling, Pan et al.^11^ used the cutting copper fiber oriented sintered heat sinks (CCFOSHS) and found that by decreasing the porosity, heat transfer efficiency of CCFOSHS was improved slightly, but pressure drops were increased, noticeably. Lin et al.^12^ used the nanofluid-cooled microchannel heat sink for the light-emitting diodes (LEDs) and concluded that nanofluids reduced the thermal resistance more than 42.4% where 0.5% TiO_2_ nanofluid improved the heat transfer efficiency up to 38.6% compared to pure water. Another study on LED cooling improvements is performed by Lin et al.^13^ using Taguchi parametric study for the micro-channel. Also, Huang and Wang^14^ found the optimal fins geometry for the circular micro-channel of LED lighting heat sinks, numerically. They found that thermal resistance of systems for the optimum design were decreased by 16.8% and 11.0% than the “initial” and “Type 2” design heat sinks, respectively.

There are also some researchers focused on the novel designs for LED heat sink applications to improve the thermal managements. Wang et al.^15^ designed novel tubular oscillating heat pipe with sintered copper particles for this application and reported that the temperature of LED array was inversely related to the illumination intensity. Tang et al.^16^ developed an integrated heat sink with vapor chamber for LED thermal managements and showed that the high-power LED yields a favorable performance using proposed unit. Kim et al.^17^ proposed the using cupper-oxide (CuO) composite coating on aluminum-alloy heat sink to enhance the heat dissipation of LED module and observed good results due to the improved thermal radiation property. In a different study, Park et al.^18,19^ designed a chimney over a circular heat sink in a downlight LED and reported that installing chimney can increase the cooling efficiency of heat sink up to 20%. Microchannels not only is applicable for LED cooling, but also they are very useful instruments for thermoelectric generators^20^, natural circulation loops^21^ and etc. which different base fluids such as PCM^22^ and nanofluids^23^ are widely used to improve their performance.

Nanofluids due to improvements thermal properties have wide applications and motivated researchers to use them in various aspects. Ben Hamida et al.^24^ used Ethylene Glycol-Copper Nanofluid under magnetic fields in an enclosure. Ben Jaballah et al.^25^ applied the hybrid nanofluid for the performance enhancement of bubble absorber for cooling applications. Hatami and Ganji^26^, Hatami et al.^27^ and Tang et al.^28^ used the nanofluid in porous media between two coaxial cylinders, wavy microchannel and wavy cavity, respectively. Also, Hatami et al.^29–32^ used the optimization techniques such as Response Surface Methodology (RSM) to find the optimize geometries including nanofluids in heat transfer applications. Massoudi et al.^33,34^ also investigated the nanofluids application in free convection under the influence of magnetic field.

Many researchers tried to find the correlations of nanofluids properties in different application. Alsarraf et al.^35^ used a multifunctional optimization for the nanofluid properties to cool the electronic heat sink through the natural convection. Also, Shahsavar et al.^36^ investigated the variable properties of Fe3O4/CNT/water hybrid nanofluid on the forced convection of mini-channel heat exchanger and found that the error of computed heat transfer rate was not exceeded tha 2.91%. Gheynani et al.^37^ studied the effect of CuO nanoparticles diameter on heat transfer on non-Newtonian nanofluid in a microtube through the changes in the thermal properties of nanofluid. Kavusi and Toghraie^38^ tested the various nanofluids (which have different thermal properties) on the performance of a heat pipe and reported that nanoparticle concentration had the greatest effect on the fluid thermal conductivity and thermal resistance. Not only different nanofluids properties were investigated by researches, but also different applications are considered for them. For instance, Gholami et al.^39^, Barnoon et al.^40^, Toghraie et al.^41^ and Arasteh et al.^42^ utilized the nanofluids and investigated their behavior under different conditions for microchannel, cavity with rotating cylinders, L-shaped porous ribs in microchannel and porous heat sink, respectively.

Based on above discussed literature review, a few studies focused on the nanofluid application for LED cooling using finned micro-channel. So, to fill this gap of study, it is tried to enhance the cooling efficiency of a LED by using the Al_2_O_3_-water nanofluid and finned arrangements in a microchannel using numerical Galerkin weighted residual Finite Element Method (GFEM), simultaneously. Also, RSM is used to find the optimized dimensions for the fin numbers, lengths and thicknesses, numerically.

## Problem description

**Figure 1:**
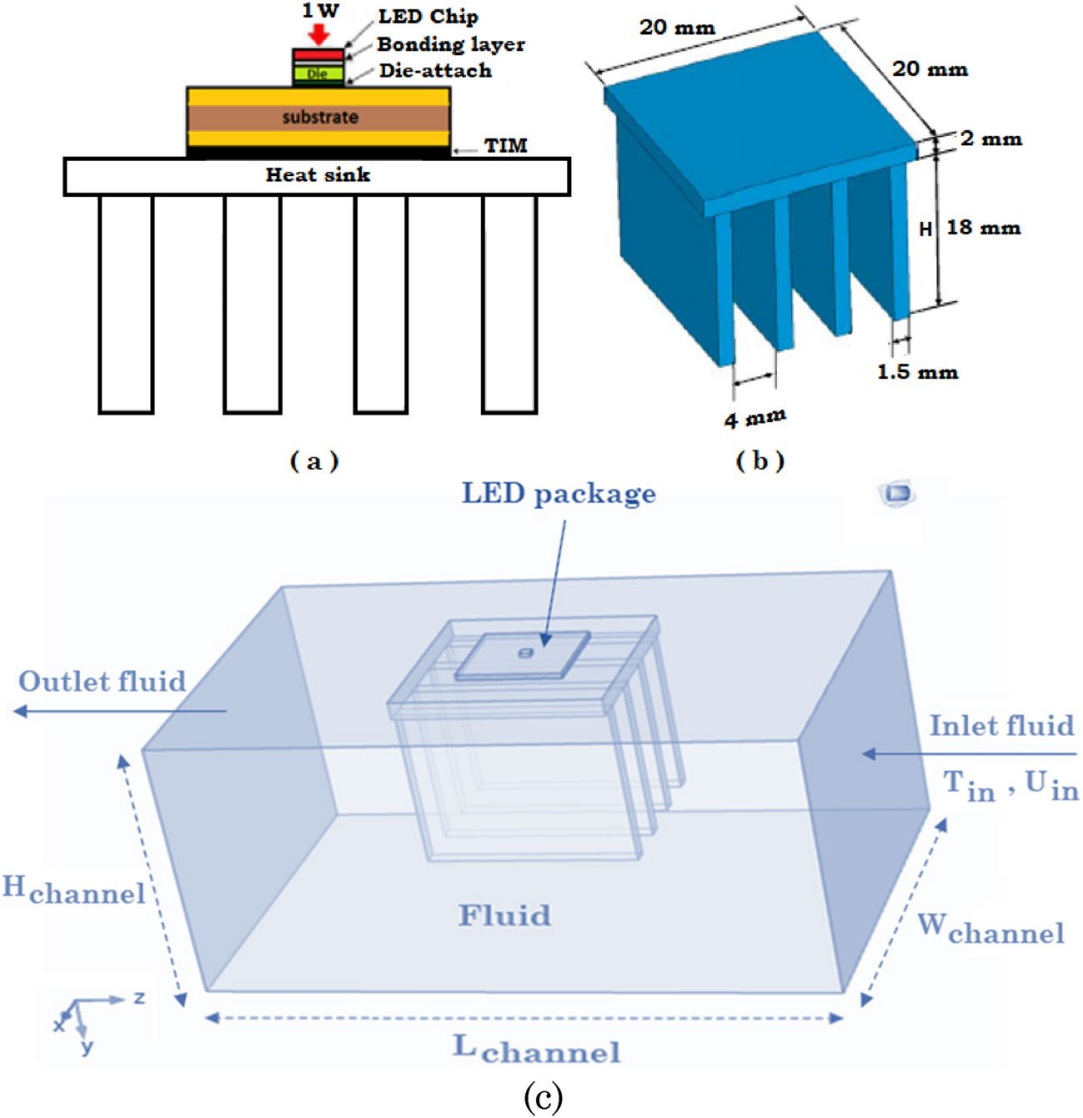
(**a**) Structure of the LED package; (**b**) Sample Dimensions of heat sink, (**c**) Geometry of LED cooling by finned micro-channel filled by nanofluid (COMSOL Multiphysics 5.6, https://www.comsol.com).

A 3D microchannel filled by Al_2_O_3_–water nanofluid is considered as shown in Fig. 1 for LED cooling by fin arrangements. Microchannel dimensions are: Height = 100 mm; Width = 80 mm and Depth = 50 mm and the Power of LED = 1, 2 and 3 W as shown in Table 1 in details. The velocity of inlet of nanofluid was considered as 0.001 m/s where the temperature of inlet of nanofluid considered to be 25 °C. The micro-channel is equipped with fin arrangements for better cooling performance. The main objective of current research is finding the optimum values for fin numbers, lengths and thicknesses, respectively as shown in Fig. 1 for the micro-channel geometry. For modeling the problem, a transient flow, incompressible three-dimensional flow from the laws of conservation of mass, momentum, and energy is considered. The governing equations represent the transient flows are^8^:

**Table 1:** Properties of dimensions and materials of LED package^8^.

	Thickness	Size	Materials
LED-chip	4 µm	1 mm × 1 mm	GaN
Metallization: Bonding Layer	10 µm		Au–Si eutectic bonding
Die	375 µm		Si
Die-attach	50 µm		Au-20Sn
Substrate	127 µm	1 cm × 1 cm	Copper
	381 µm		AlN
TIM	50 µm	1 cm × 1 cm	Thermal grease
Heat sink	–	**–**	Al

Mass conservation equation:

1 $$\frac{\partial \rho }{{\partial t}} + \frac{{\partial (\rho {\kern 1pt} u)}}{\partial x} + \frac{{\partial (\rho {\kern 1pt} v)}}{\partial y} + \frac{{\partial (\rho {\kern 1pt} w)}}{\partial z} = 0$$

where ρ refers to the mass density and u, v, and w represent the velocities according to x, y, and z, respectively.

Momentum conservation equation according to x:

2 $$\frac{\partial (\rho \,u)}{{\partial t}} + \frac{{\partial (\rho {\kern 1pt} u{\kern 1pt} u)}}{\partial x} + \frac{{\partial (\rho {\kern 1pt} u{\kern 1pt} v)}}{\partial y} + \frac{{\partial (\rho {\kern 1pt} u{\kern 1pt} w)}}{\partial z} = - \frac{\partial p}{{\partial x}} + \left[ {\frac{\partial }{\partial x}(\eta \frac{\partial u}{{\partial x}}) + \frac{\partial }{\partial y}(\eta \frac{\partial u}{{\partial y}}) + \frac{\partial }{\partial z}(\eta \frac{\partial u}{{\partial z}})} \right]$$

Momentum conservation equation according to y:

3 $$\frac{{\partial (\rho {\kern 1pt} v)}}{\partial t} + \frac{{\partial (\rho {\kern 1pt} u{\kern 1pt} v)}}{\partial x} + \frac{{\partial (\rho {\kern 1pt} v{\kern 1pt} v)}}{\partial y} + \frac{{\partial (\rho {\kern 1pt} v{\kern 1pt} w)}}{\partial z} = - \frac{\partial p}{{\partial y}} + \left[ {\frac{\partial }{\partial x}(\eta \frac{\partial v}{{\partial x}}) + \frac{\partial }{\partial y}(\eta \frac{\partial v}{{\partial y}}) + \frac{\partial }{\partial z}(\eta \frac{\partial v}{{\partial z}})} \right] + \rho {\kern 1pt} g$$

Momentum conservation equation according to z:

4 $$\frac{{\partial (\rho {\kern 1pt} w)}}{\partial t} + \frac{{\partial (\rho {\kern 1pt} u{\kern 1pt} w)}}{\partial x} + \frac{{\partial (\rho {\kern 1pt} v{\kern 1pt} w)}}{\partial y} + \frac{{\partial (\rho {\kern 1pt} w{\kern 1pt} w)}}{\partial z} = - \frac{\partial p}{{\partial z}} + \left[ {\frac{\partial }{\partial x}(\eta \frac{\partial w}{{\partial x}}) + \frac{\partial }{\partial y}(\eta \frac{\partial w}{{\partial y}}) + \frac{\partial }{\partial z}(\eta \frac{\partial w}{{\partial z}})} \right]$$

In above equations, p denotes the pressure, g shows the gravity, and η is related to the dynamic viscosity.

Energy conservation equation:

5 $$\frac{{\partial (\rho {\kern 1pt} C{\kern 1pt} T)}}{\partial t} + \frac{{\partial (\rho {\kern 1pt} u{\kern 1pt} C{\kern 1pt} T)}}{\partial x} + \frac{{\partial (\rho {\kern 1pt} v{\kern 1pt} {\kern 1pt} C{\kern 1pt} T)}}{\partial y} + \frac{{\partial (\rho {\kern 1pt} w{\kern 1pt} \,C{\kern 1pt} T)}}{\partial z} = \left[ {\frac{\partial }{\partial x}(\lambda \frac{\partial T}{{\partial x}}) + \frac{\partial }{\partial y}(\lambda \frac{\partial T}{{\partial y}}) + \frac{\partial }{\partial z}(\lambda \frac{\partial T}{{\partial z}})} \right]$$

where, T and C are the temperature and the specific heat capacity, respectively and λ is the thermal conductivity.

The practical boundary conditions are assumed as follows:

- There is a uniform heat flux on top of the die, and other surfaces are adiabatic.
- The microchannel inlet boundary condition is considered to be the uniform temperature of T_in_ with a uniform velocity of U_in_.
- At microchannel outlet, Pressure outlet boundary condition with zero gradients is assumed.
- No slip condition is imposed on the surface of microchannel walls.
- All the microchannel outer walls are insulated.

In this study, the thermal conductivity of the materials used in the LED package as well as the water properties are considered dependent on temperature^8^. For water temperature-dependent properties:

(A) Dynamic viscosity:

273.15 K < T < 473.15 K:

6 $$\eta = { 1}.{37995668}0{4 } - \, 0.0{21224}0{19151}{*} {\text{T }} + { 1}.{36}0{4562827}{*} {1}0^{{ - {4}}}{*} {\text{T}}^{{2}} - { 4}.{6454}0{9}0{319}{*} {1}0^{{ - {7}}}{*} {\text{T}}^{{3}} + { 8}.{9}0{42735735}{*} {1}0^{{ - {1}0*}} {\text{T}}^{{4}} - { 9}.0{79}0{692686}{*} {1}0^{{ - {13}}}{*} {\text{T}}^{{5}} + { 3}.{8457331488}{*} {1}0^{{ - {16}}}{*} {\text{T}}^{{6}}$$

473.15 < T < 553.15 K:

7 $$\eta = 0.00{4}0{1235783 } - { 2}.{1}0{746715}*{1}0^{{ - {5}}} *{\text{T }} + { 3}.{85772275}*{1}0^{{ - {8}}} *{\text{T}}^{{2}} - { 2}.{3973}0{284}*{1}0^{{ - {11}}} *{\text{T}}^{{3}}$$

T in K and dynamic viscosity in Pa.s

(B) Thermal capacity:

273.15 < T < 553.15 K

8 $${\text{C }} = 12010.1471 - 80.4072879*{\text{T }} + 0.30866854*{\text{T}}^{{2}} - 5.38186884*10^{{- {4}}} *{\text{T}}^{{3}} + 3.62536437*10^{-7} *{\text{T}}^{{4}}$$

T in K and Thermal Capacity in J/(kg*K).

(C) Mass density

273.15 < T < 293.15 K.

9 $$\rho = 0.0000{63}0{92789}0{34}*{\text{T}}^{{3}} - 0.0{6}0{367639882855}*{\text{T}}^{{2}} + {18}.{92293824}0{7}0{66}*{\text{T }} - {95}0.{7}0{4}0{55329848}$$

293.15 < T < 373.15 K.

10 $$\rho = 0.0000{1}0{335}0{53319}*{\text{T}}^{{3}} - 0.0{13395}0{65634452}*{\text{T}}^{{2}} + {4}.{96928883265516}0*{\text{T}} + { 432}.{257114}00{8512}$$

T in K and mass density in kg/m^3^.

(D) Thermal conductivity

273.15 < T < 1000 K

11 $$\lambda = - 0.{869}0{83936} + 0.00{89488}0{345}{*}{\text{T}} - {1}.{58366345}{*}{1}0^{{ - {5}}}{*}{\text{T}}^{{2}} + {7}.{97543259}{*}{1}0^{{ - {9}}}{*}{\text{T}}^{{3}}$$

T in K and Thermal conductivity in W/(m*K). For the nanofluid properties, following equations are applied^35^:

12 $$\rho_{nf} = \left( {{1} - \phi } \right)\rho_{bf} + \varphi \rho_{p}$$

13 $${C}_{{p}_{nf}}=\frac{\left(1-\varphi \right){\left(\rho {C}_{p}\right)}_{bf}+\varphi {(\rho {C}_{p})}_{p}}{{\rho }_{nf}}$$

14 $$\begin{gathered} \frac{{k_{nf} }}{{k_{bf} }} = 0.991 + 0.276T\varphi + 77.6\varphi^{2} + 3641.231T\varphi^{2} + \hfill \\ \frac{0.00217}{{\sin \left( {T - \varphi } \right)}} - 6.01 \times 10^{ - 6} T^{2} - 3647.099T\varphi \sin \left( \varphi \right) \hfill \\ \end{gathered}$$

15 $$\mu_{nf} = \mu_{static} + \mu_{Brownian}$$

16 $$\mu_{nf} = \frac{{\mu_{f} }}{{\left( {1 - \varphi } \right)^{2.5} }} + 5 \times 10^{4} \beta \varphi \rho_{f} \left( {C_{p} } \right)_{f} \frac{{\mu_{f} }}{{k_{f} \Pr }}\sqrt {\frac{{k_{b} T}}{{\rho_{p} d_{s} }}} f\left( {T,\varphi } \right)$$

where

17 $$f\left( {T,\varphi } \right) = \left( {2.8217 \times 10^{ - 2} \varphi + 3.917 \times 10^{ - 3} } \right)\left( {\frac{T}{{T_{0} }}} \right) + \left( { - 3.0669 \times 10^{ - 2} \varphi - 3.91123 \times 10^{ - 3} } \right)$$

18 $$\beta = 8.4407\left( {100\varphi } \right)^{ - 1.07304}$$

## Methodology of solution

In this study, COMSOL-Multiphysics commercial Galerkin finite element method (GFEM) software is applied for the modeling of problem. GFEM is a numerical method to discretize and solve the coupled partial differential equations governed on any physical phenomenon. GFEM by COMSOL is not only accurate and adaptable, but also it is simple in modeling the complicated physics as well as the time-dependent simulation of physical problems in environment, chemical, energy, electrical, heat transfer and other multi-physic problems. Actually, GFEM with COMSOL can help the user to solve the problems with multi-physics such as electrical–mechanical systems easily, while the solution of these problems by other commercial software (such as FVM based ANSYS-FULENT) or by hand is not possible^8^. During the modeling process, user must define the boundary conditions after the geometry modeling to show the conditions which must be responded during the solution. Boundary conditions can be defined as the distributed forces, point forces, positional constraints and thermal effects such as temperature changes or applied heat energy. Additionally, the GFEM user can certainly spot any vulnerability in design with the complete visualizations GFEM produces and then use the novel data to have a new design^8^.

Central Composite Design or CCD is a technique of Design of Experiment (DoE) to achieve the suitable points of each independent variable based on their possible values (level). CCD contains an imbedded factorial or fractional factorial design with center points which is improved by a “star points” group and let’s estimate of curvature. After introducing the minimum number of cases with CCD, RSM must be applied to find the optimum values for the fin numbers, lengths and thicknesses. Actually, RSM find a first or second-order polynomial relation between the response (here temperature) and independent variables (fin numbers, lengths and thicknesses). This polynomial equation consider the interaction between the and surface curvatures and fitted in the shape of^31,32^

19 $$y = A_{0} + \sum\limits_{i = 1}^{n} {A_{i} x_{i} } + \sum\limits_{i = 1}^{n} {A_{ii} x_{i}^{2} } + \sum\limits_{i = 1}^{n} {\sum\limits_{j = 1}^{n} {\left. {A_{ij} x_{i} x_{j} } \right|_{i < j} } }$$

where *x*_*i*_ and *x*_*j*_ indicate the geometry independent variables (fin numbers, lengths and thicknesses) and *A* is the coefficient of polynomial equation. The essential parameter for RSM optimization is “Desirability” which returns the desirable ranges of responses (d_i_). Actually, objective function can be altered from zero outside of the limits to unity at the aim and the point with the maximum desirability function by following equation is the optimized case obtained by numerical solutions:

20 $$D = \left( {d_{1} \times d_{2} \times ... \times d_{n} } \right)^{\frac{1}{n}} = \left( {\prod\limits_{i = 1}^{n} {d_{i} } } \right)^{\frac{1}{n}}$$

where *n* indicates the number of responses. If each of responses or factors drops outside their desirability range, the overall function will be zero. More details about this optimization method can be found in^31,32^.

## Results and discussions

As described in above sections, it is tried to obtain an optimized geometry for the microchannel filled by nanofluid for LED cooling. Dimensions of LED package as well as the materials are presented in Table 1. Based on previous study in^8^, the best materials for LED-chip is GaN, Die from Si, Die-attach is made by Au-20Sn, substrate is copper and heat sink material is considered to be Al. LED has 1, 2 and 3 W power and the microchannel dimensions was Height = 100 mm; Width = 80 mm and Depth = 50 mm. In order to find a suitable mesh grid, numerous mesh numbers (from extremely coarse to finer mesh) were used for this 3D model as demonstrated in Table 2. These values confirm that fine mesh is a suitable grid type for this study due to its accuracy and acceptable time of calculations. Then, CCD is applied to have the proposed geometries for the considered parameters as shown in Table 3. Fin number, fin diameter and fin thicknesses are three main parameters under the study which their levels were presented in Table 3. CCD proposed 11 cases as the minimum required cases which are shown in Table 4 by details.

**Table 2:** Mesh independency of the micro-channel.

Predefined mesh size	Mesh elements	Junction Temperature (°C) (max. temperature)	Temperature of Heat sink (°C) (min. temperature)
Extremely coarse	3641	60.165	52.416
Extra coarse	6019	60.231	52.623
Coarser	10,766	60.254	52.794
Coarse	17,989	60.273	52.931
Normal	26,436	60.289	53.087
Fine	45,255	60.370	53.222
Finer	136,857	60.968	53.568

**Table 3:** Geometry parameters of fins arrangement for microchanel and defined levels.

Parameter	Description	Level 1	Level 2	Level 3
A	Fin number	4	6	8
B	Fin Length	10	15	20
C	Fin thickness	2	3	4

**Table 4:** CCD proposed cases for finned geometry with Power of LED = 1, 2 and 3 W Microchannel dimensions: Height = 100 mm; Width = 80 mm and Depth = 50 mm.

Case number	Level of parameters		
	Parameter, A	Parameter, B	Parameter, C
1	6	20	3
2	4	15	3
3	4	10	2
4	6	15	3
5	4	20	4
6	8	10	4
7	6	15	2
8	8	15	3
9	8	20	2
10	6	10	3
11	6	15	4

**Figure 2:**
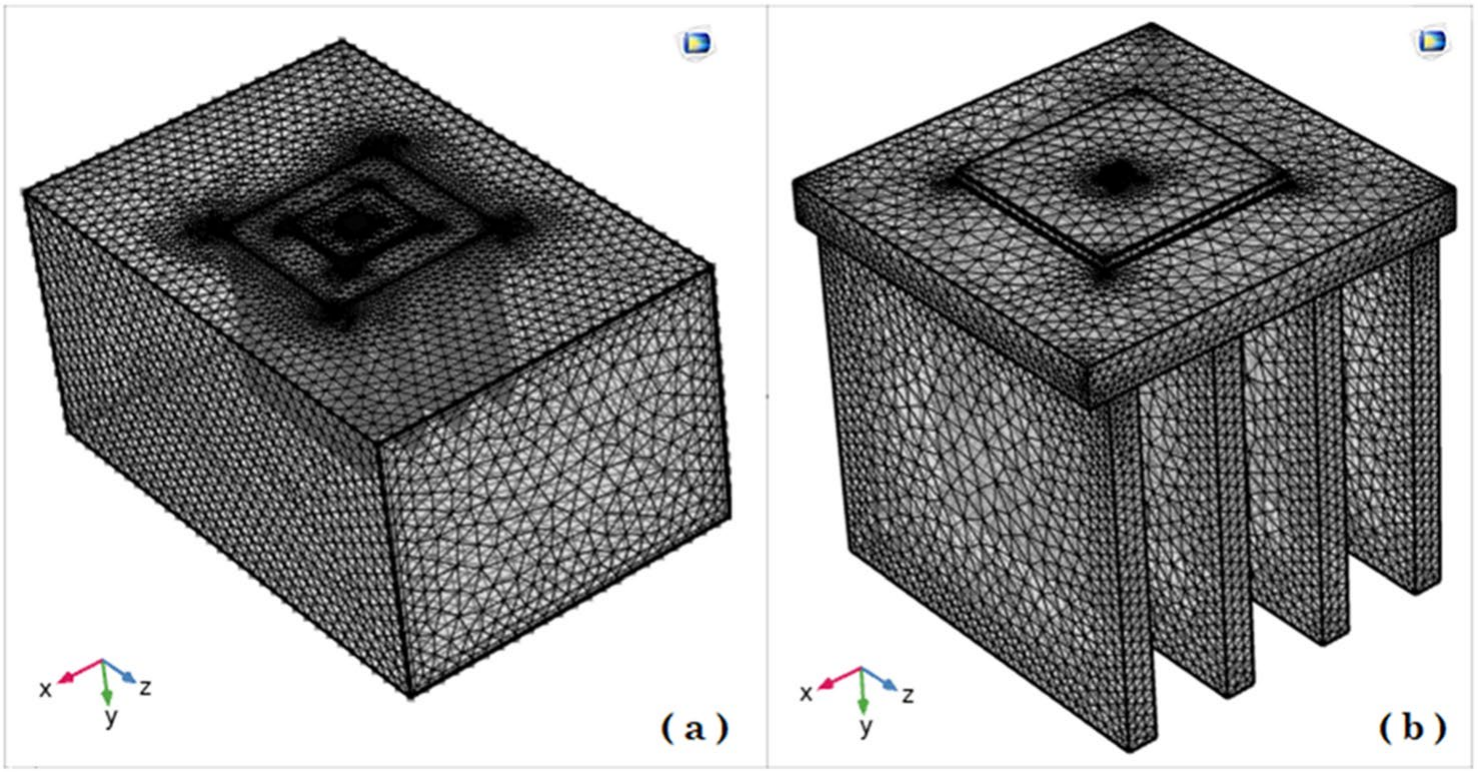
Mesh generated in the domain and LED finned section (COMSOL Multiphysics 5.6, https://www.comsol.com).

**Figure 3:**
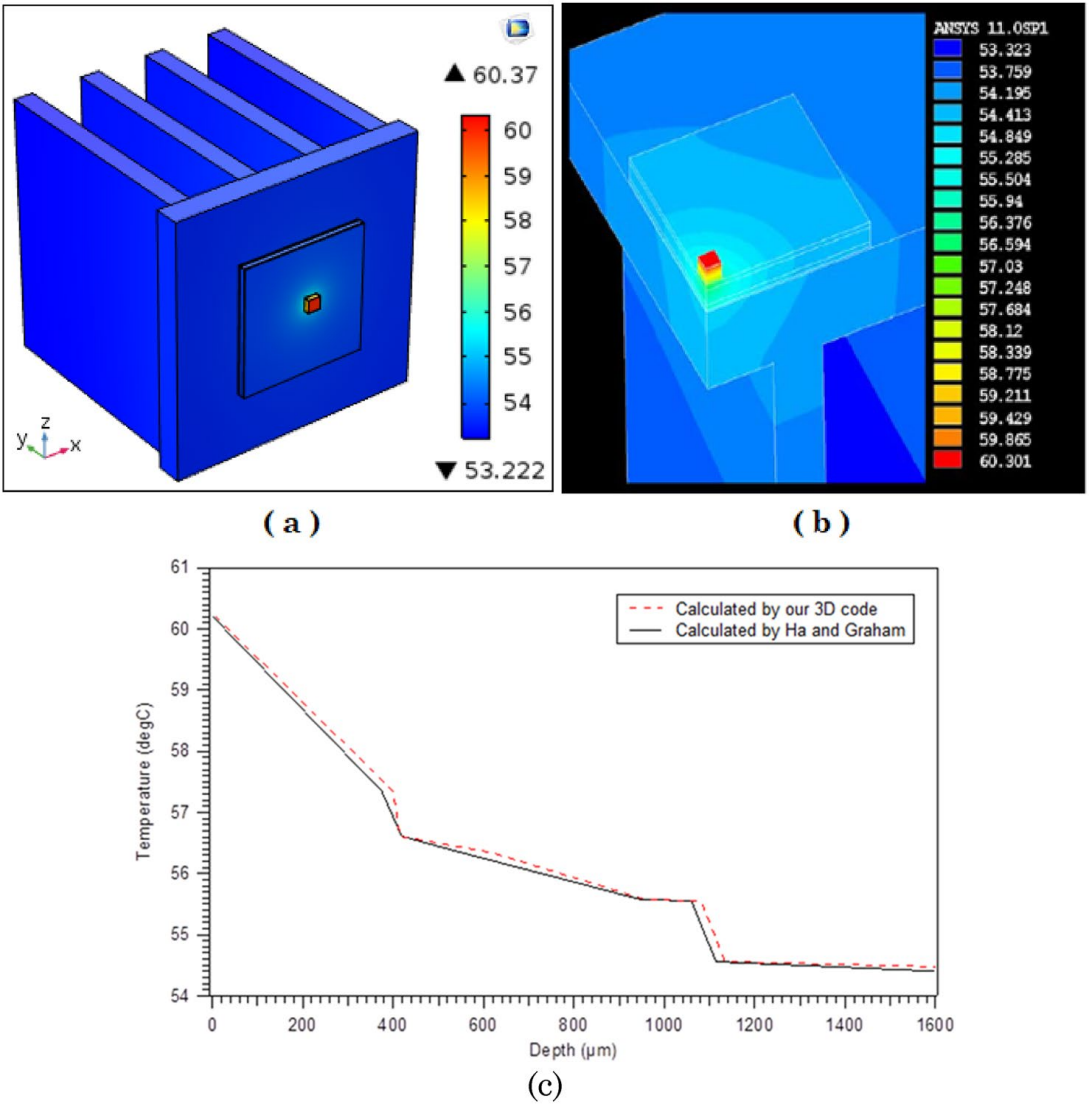
Temperature distribution of the LED package: (**a**): Our 3D code (**b**): Ha and Graham code^7^ (**c**), Comparison of vertical temperature profile along the center line (z axis)^8^ (COMSOL Multiphysics 5.6, https://www.comsol.com).

To validate our generated 3D model with the previous model of Ha and Graham^7^, the temperature distribution of the LED package is simulated with the equivalent settings modeled by Ha and Graham^7^ as presented in Fig. 1 and generated mesh is depicted in Fig. 2. Figure 3 shows the 3D model simulated by Ha and Graham^7^ using the commercial ANSYS-FLUENT software where Power of LED lamp was 1 W, heat transfer coefficient was 10 W/m^2^/K and the temperature of ambient was 25 °C. From the Fig. 3a,b it can be observed that current code which also was used in^8^ has similar results with^7^ where the minimum and maximum temperatures for both methods are about 53.22 °C and 60.37 °C, respectively. Actually a minor temperature difference between these two models is existed which does not exceed than 0.001%. Also, the vertical temperature profile along the LED package centerline is plotted in Fig. 3c, which a good agreement between current 3D COMSOL-Multiphysics model and 3D ANSYS-FLUENT model of Ha and Graham^7^ was observed.

**Figure 4:**
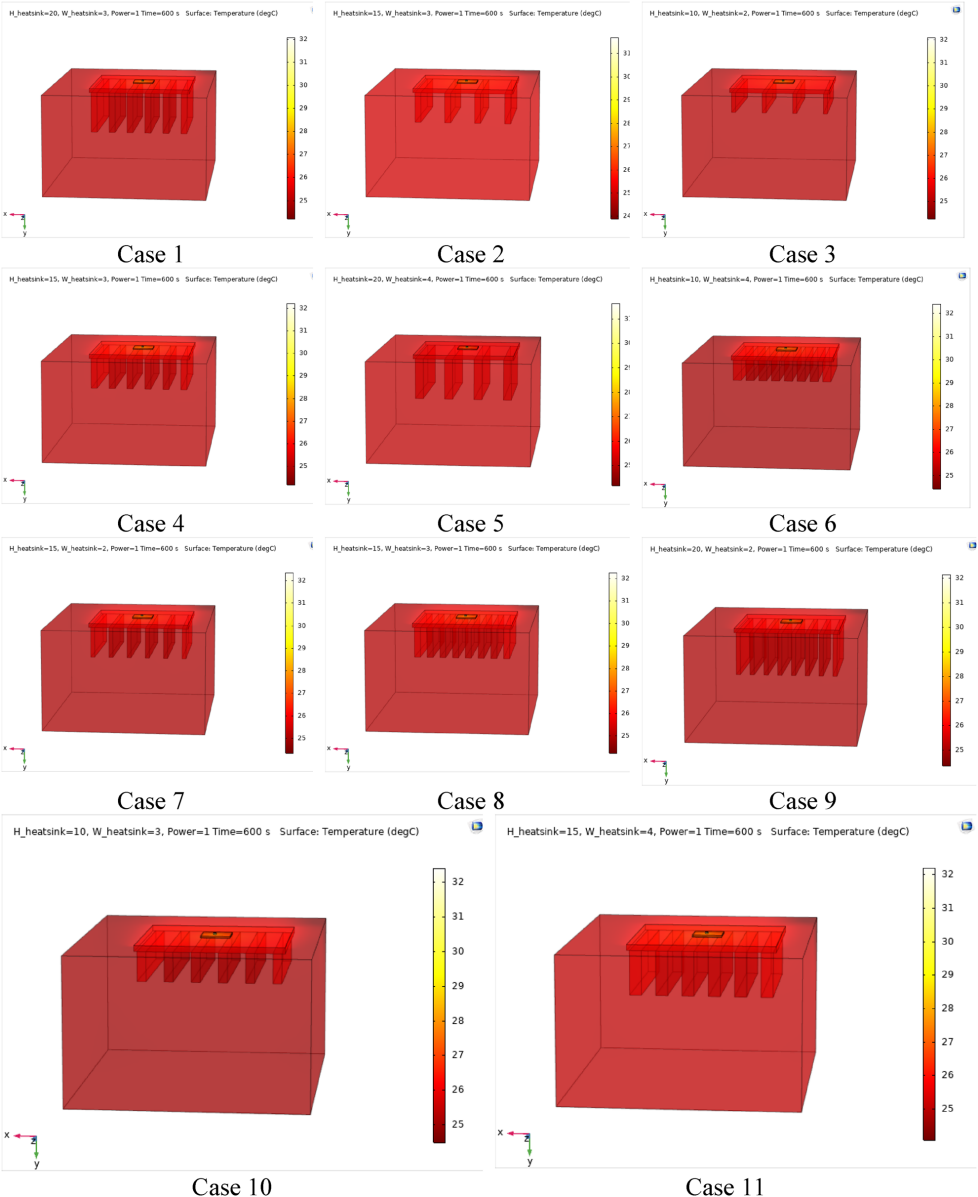
Temperature contours for the finned micro-channel of different geometries (11 Cases) (COMSOL Multiphysics 5.6, https://www.comsol.com).

**Figure 5:**
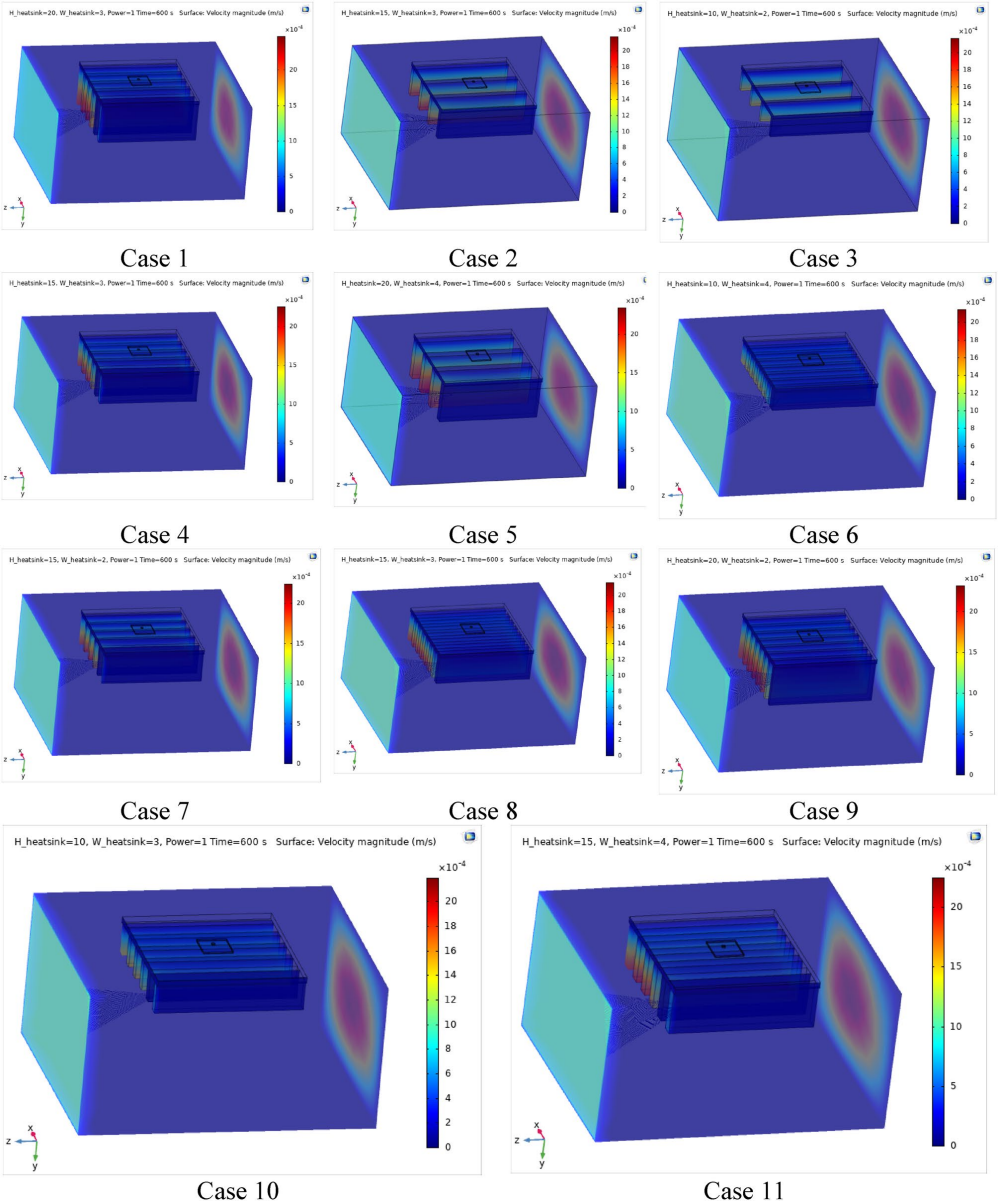
Velocity contours for the finned micro-channel of different geometries (11 Cases) (COMSOL Multiphysics 5.6, https://www.comsol.com).

**Figure 6:**
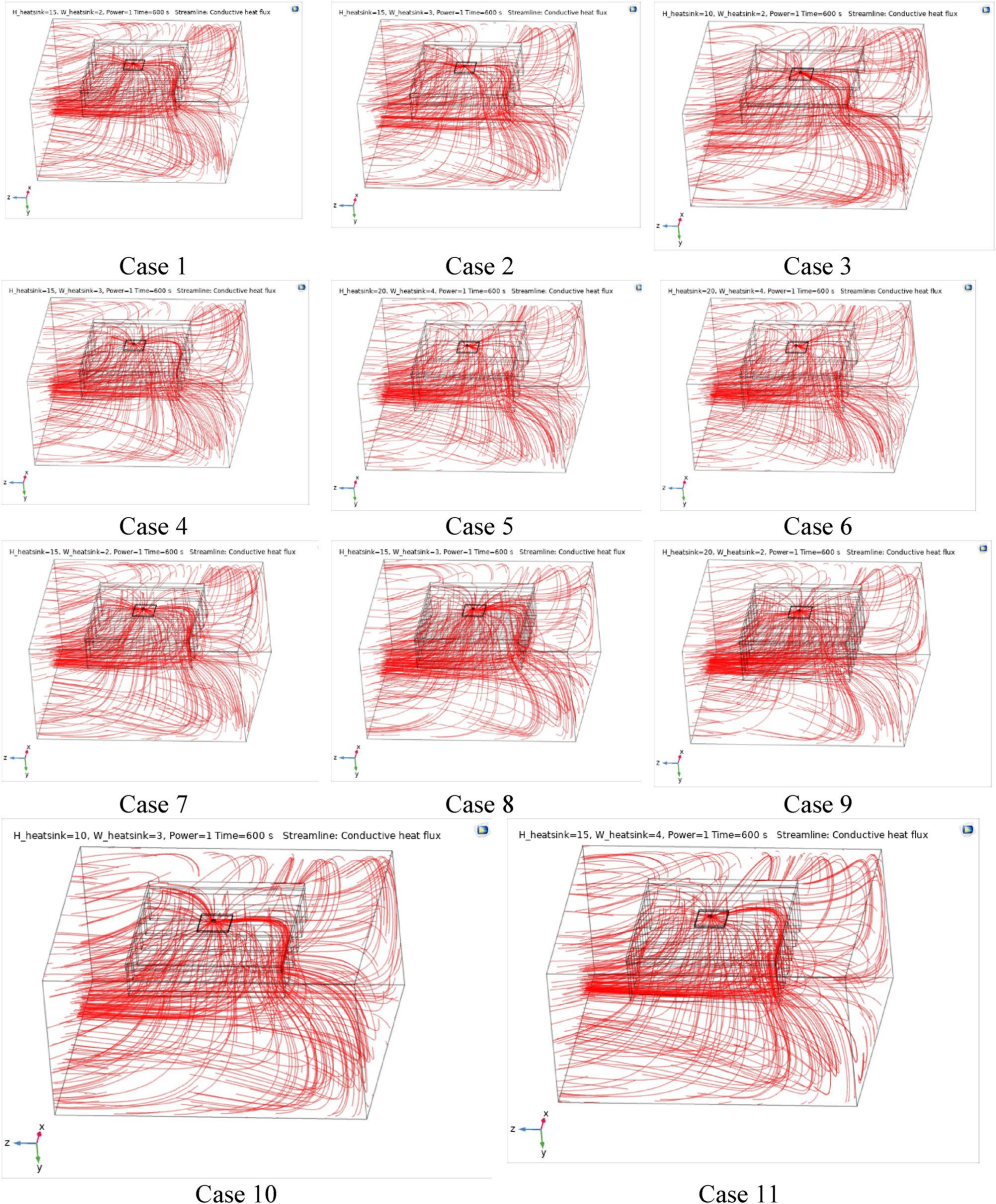
Streamline contours for the finned micro-channel of different geometries (11 Cases) (COMSOL Multiphysics 5.6, https://www.comsol.com).

**Figure 7:**
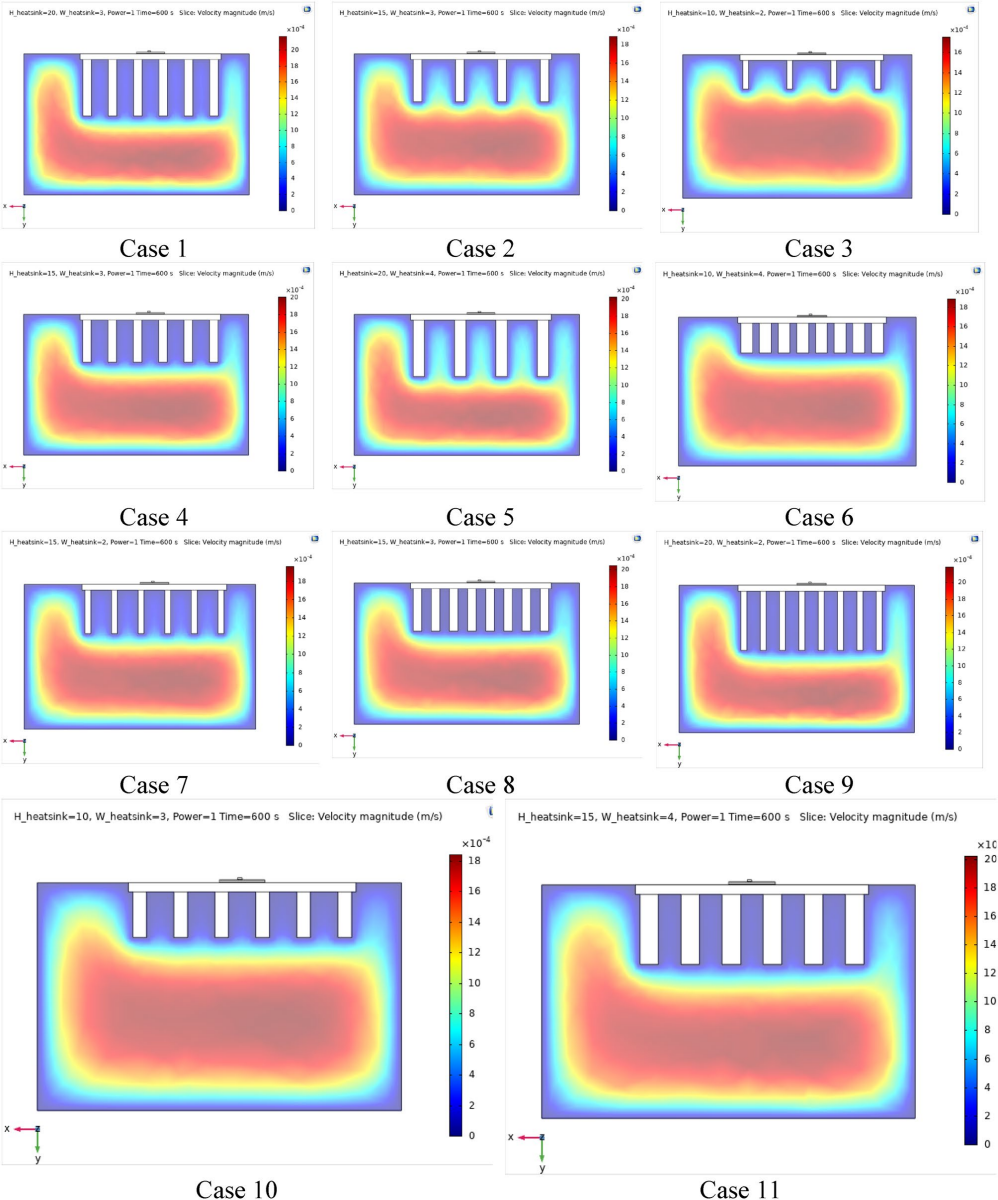
2D Velocity contours for the finned micro-channel of different geometries (11 Cases) (COMSOL Multiphysics 5.6, https://www.comsol.com).

**Figure 8:**
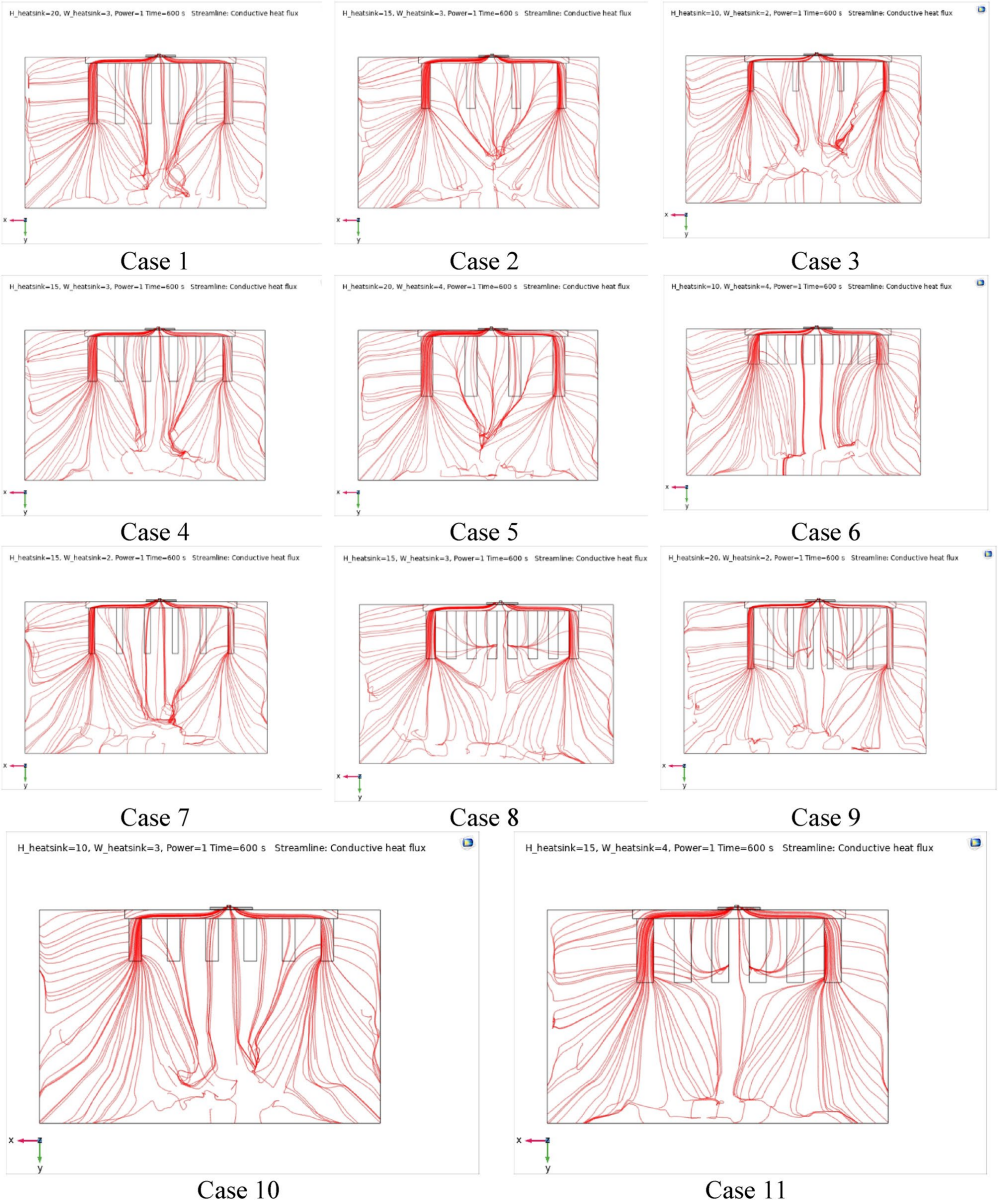
2D Streamline contours for the finned micro-channel of different geometries (11 Cases) (COMSOL Multiphysics 5.6, https://www.comsol.com).

**Figure 9:**
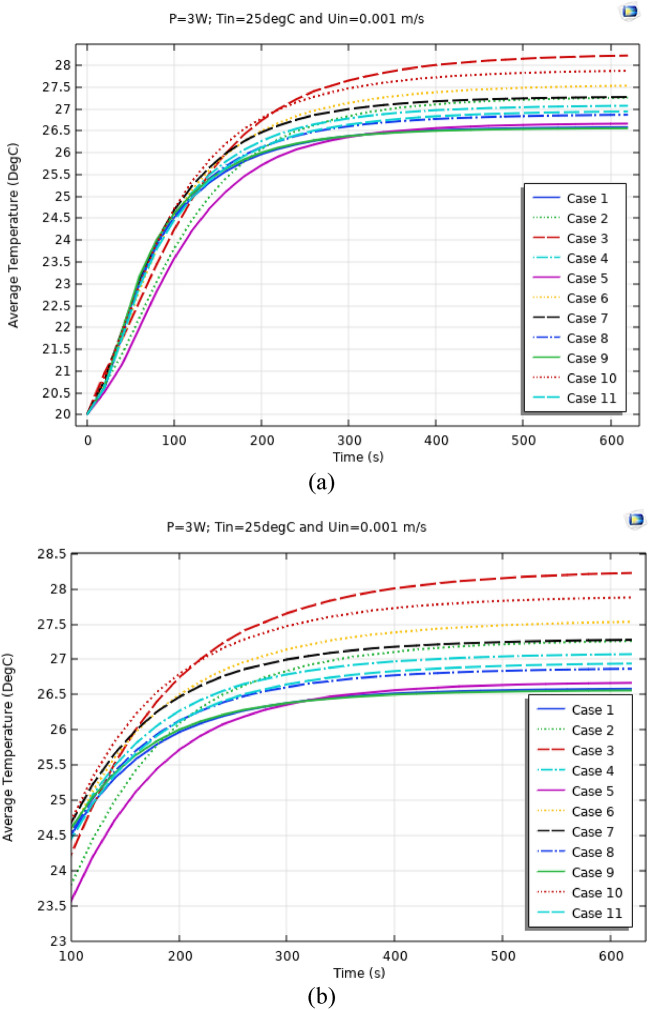
Average Temp. when P = 3 W for different 11 cases (**a**) complete view, (**b**) zoomed view.

**Figure 10:**
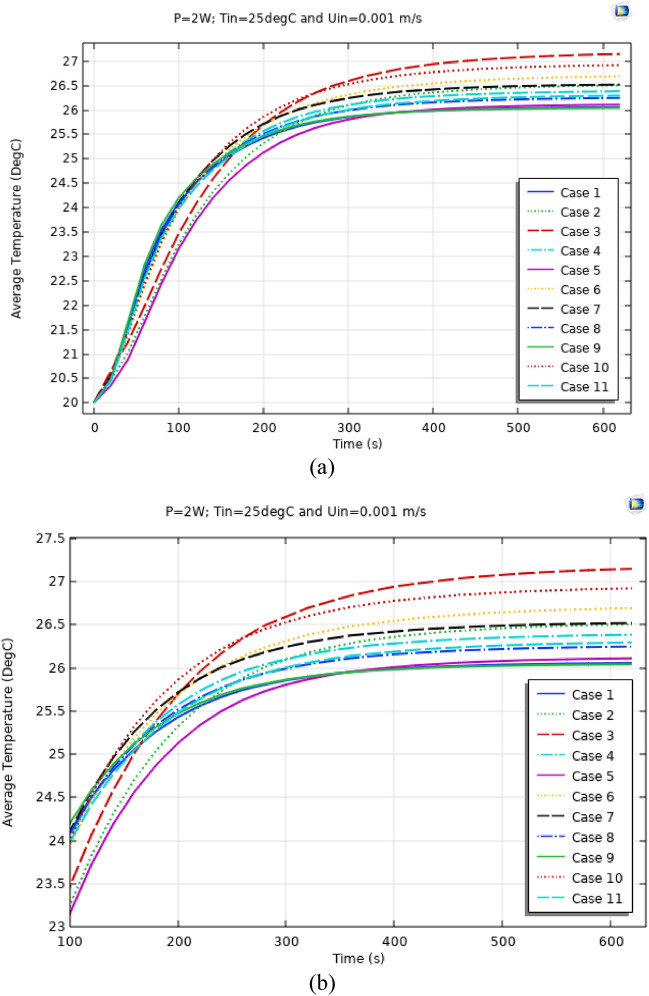
Average Temp. when P = 2 W for different 11 cases (**a**) complete view, (**b**) zoomed view.

**Figure 11:**
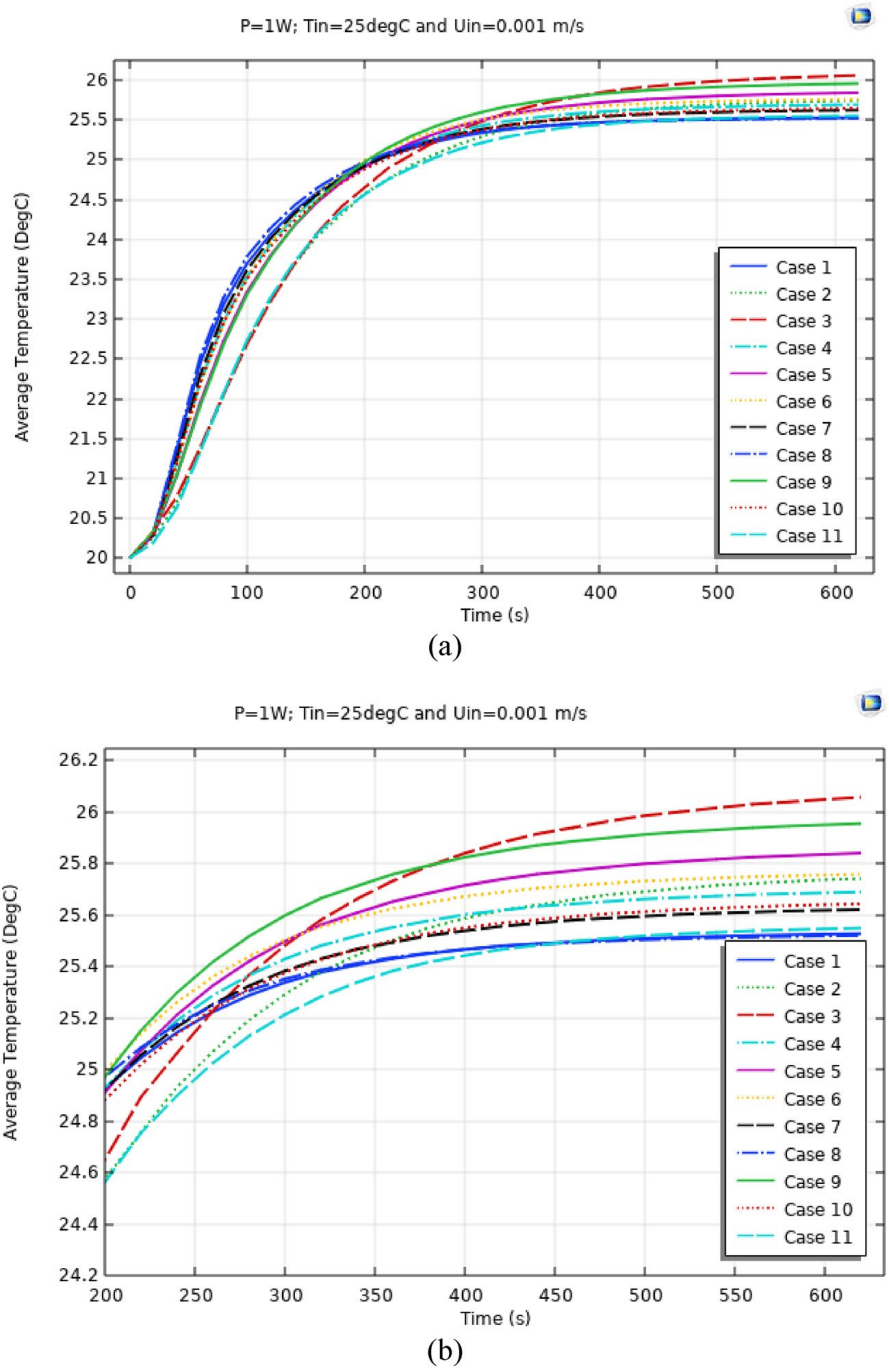
Average Temp. when P = 1 W for different 11 cases (**a**) complete view, (**b**) zoomed view.

**Figure 12:**
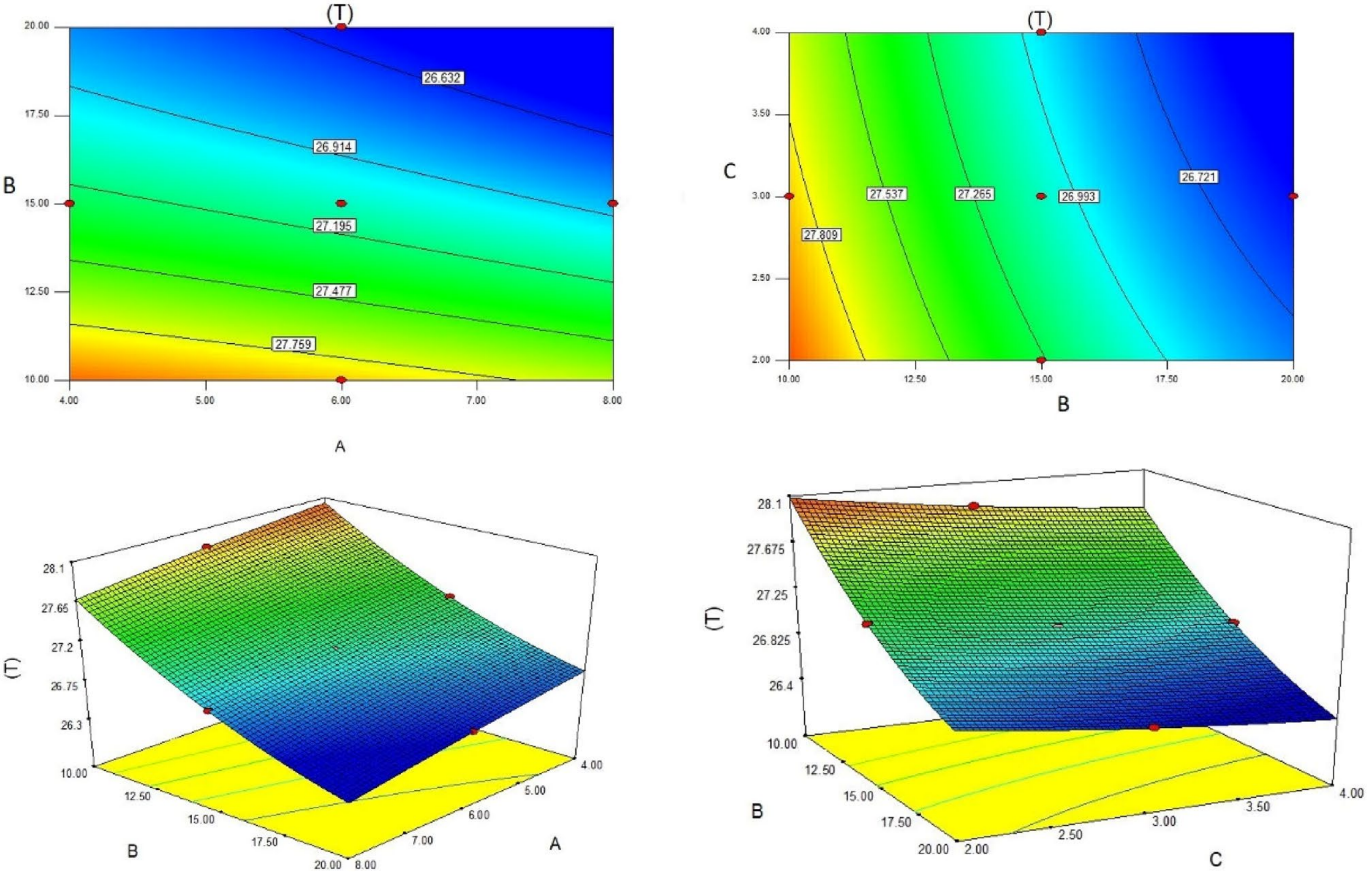
2D and 3D contour of geometry parameters on temperature.

**Figure 13:**
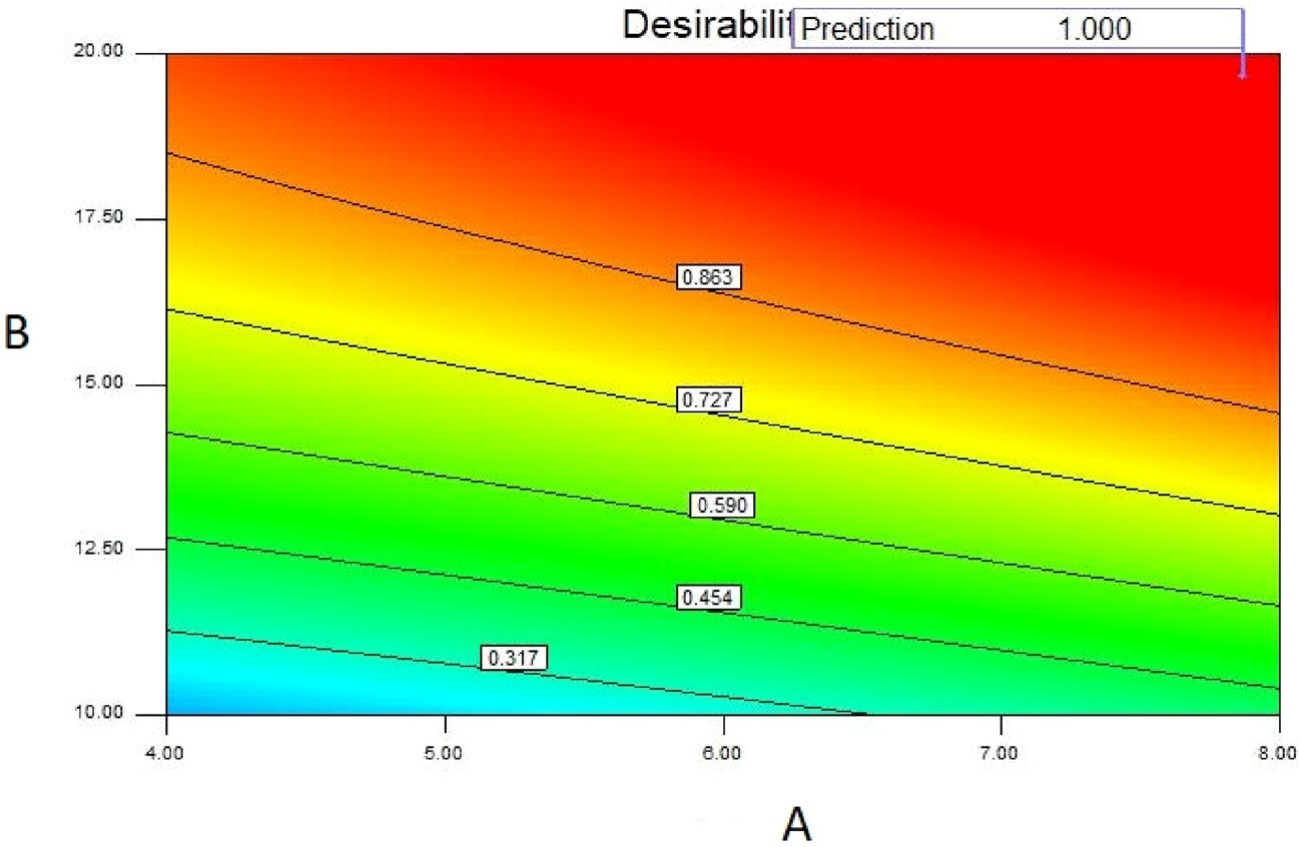
Desirability function of optimized geometry.

**Figure 14:**
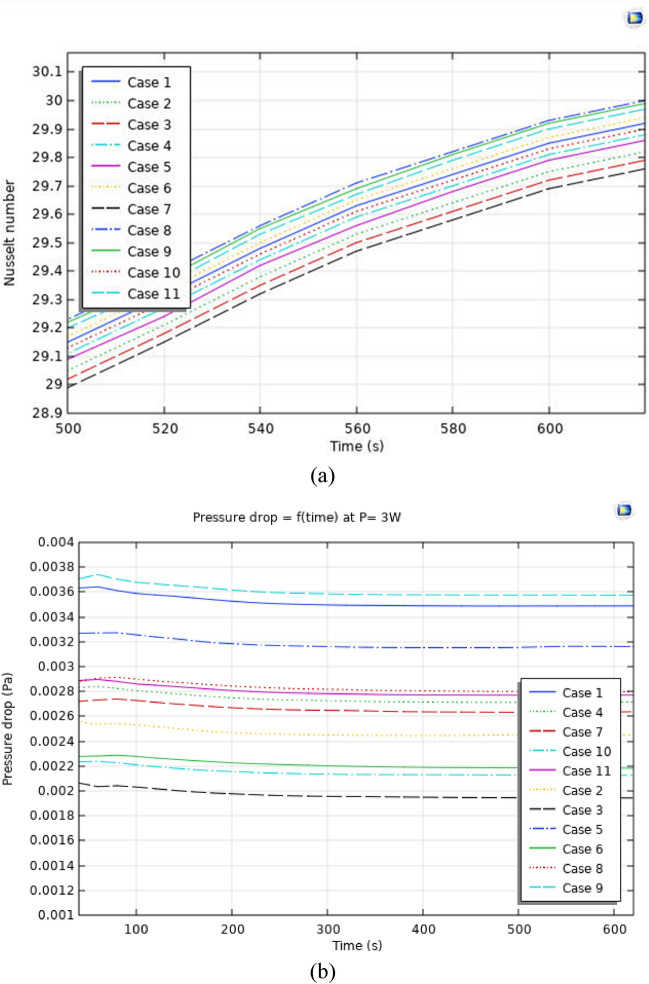
(**a**) Nusselt number (**b**) Pressure drops graphs for P = 3 W for all designed cases.

**Figure 15:**
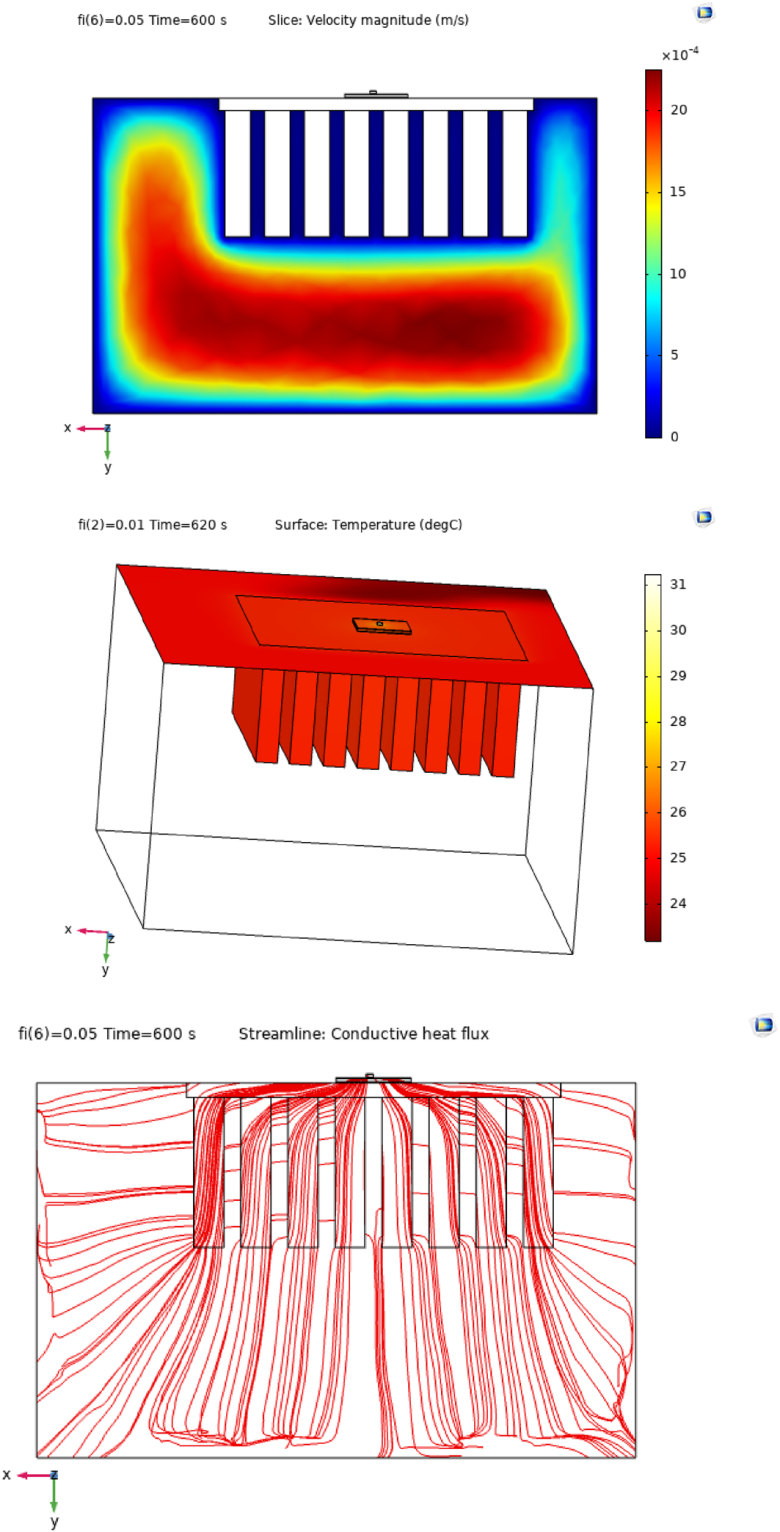
Velocity, temperature and streamline for optimized case (COMSOL Multiphysics 5.6, https://www.comsol.com).

**Figure 16:**
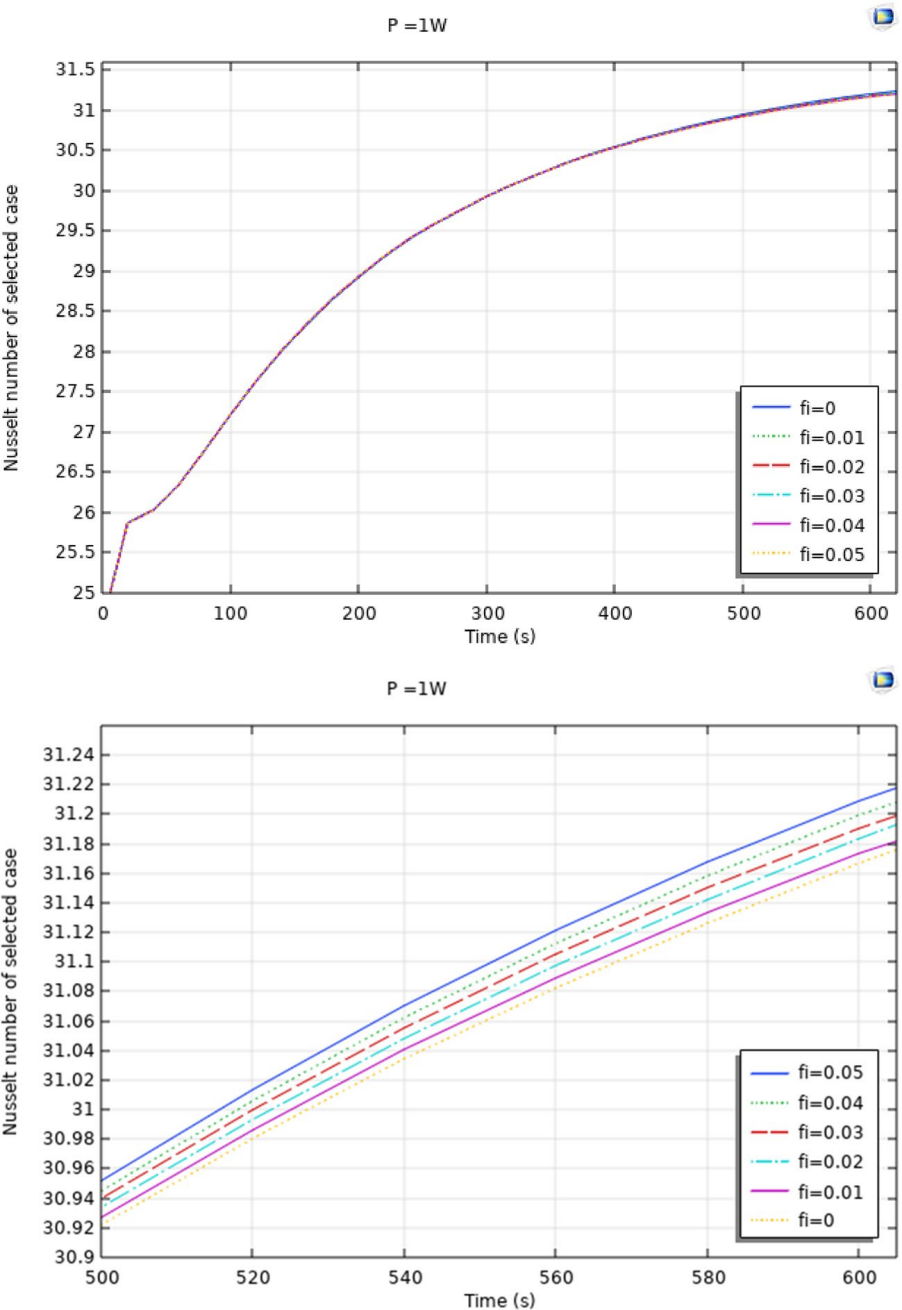
Effect of different nanoparticles concentration on Nusselt number of the optimized case.

Figures 4, 5 and 6 show the temperatures, velocities and streamlines of nanofluid in both LED and microchannel domains and the results of junction temperature for three LED powers (1, 2 and 3 W) of all 11 designed cases are shown in Table 5 at final time, t = 620 s. It is obvious that when the number of fins increased the cooling process is better occurred and maximum temperature on the LED is decreased. Figures 7 and 8 shows the 2D contours of velocity and streamlines of 11 cases, respectively. It can be observed that cases 6, 8 and 9 due to greater fin numbers (8 fins) has more compact microchannel and the velocity between the fins is greater. But for the cases 2, 3, 5 and 10 nanofluids slowly flow between the fins due to more free spaces. So, this parameter has an important role in heat transfer due to effect on both heat transfer surface and changing the flows regime. Also, the cases which have lengthy fins (Cases 1, 5 and 9) have smaller region of maximum velocity in micro-channel (red area) due to making obstacles against the flow. Because these three parameters has influence on each other, a parametric study is required to find the most important parameter among them. Figure 9 shows the average temperatures during the time for P = 3 W. Cases 3, 10 and 6 have maximum temperatures (worst cooling performance) and cases 9, 1 and 5 have the minimum temperatures or best cooling performances. From Table 4, it can be seen that Cases 3, 10 and 6 are in lowest level of fin length, while cases 9, 1 and 5 are in upper level of fins length. Figures 10 and 11 also present the temperatures during the time when LED power is 2 W and 1 W, respectively. These figures confirm the same results of cooling the LED by finned microchannel. Also, it can be found that number of fins is another important parameter while the fins thickness is not very significant compared to two other parameters due to its small effect of the cooling performance resulted from small effect on both surface and flow regime. From this figures, it can be concluded that by different designs of fins arrangements, it is possible to increase the nanofluid temperature up to 6.5% which enhanced the LED cooling process. Figure 12 shows these descriptions in 2D and 3D contours to find the effect of each parameter on the results. As seen, slope of curves and surfaces for the parameter *B* is greater than parameter *A* and greater than parameter *C*. Also the minimum temperatures (best cooling) is occurred when all the parameters are in upper levels, so desirability function of optimized case in Fig. 13 is depicted which illustrate that better case (desirability = 1) occurs when B = 20 and A = 8. Based on these results, RSM proposed the best cases which is presented in Table 6 as optimized geometries. Figure 14 compares the Nusselt number and pressure drops of all cases. Since the pressure drops are very small, so it can be negligible in optimization and single objective is performed for this case of study. The results of optimized case is depicted in Fig. 15, while the effect of different nanoparticles volume fraction is presented in Fig. 16 which says that larger nanoparticles volume fraction has greater Nusselt number due to temperature dependent properties of base water and greater thermal conductivity of nanofluid as well as the lower pressure drop for smaller nanoparticles concentrations.

**Table 5:** Results of final temperature (t = 620 s) for designed geometries.

Case	P = 1 W	P = 2 W	P = 3 W
1	25.52701	26.05767	26.58474
2	25.74088	26.50567	27.26295
3	26.05708	27.14766	28.22671
4	25.68918	26.38428	27.07537
5	25.83996	26.11025	26.66800
6	25.75795	26.69111	27.53490
7	25.62041	26.52071	27.27858
8	25.51977	26.24666	26.86830
9	25.95494	26.04184	26.56050
10	25.64296	26.92147	27.88059
11	25.54928	26.29494	26.94251

**Table 6:** Optimized geometry proposed by RSM.

Number	A	B	C	
1	**8**	**20**	**4**	**Selected**
2	8	19.64	3.89	
3	6	19.83	3.17	
4	8	18.84	2.63	
5	8	19.06	2.51

## Conclusion

In this study, the geometry of a finned microchannel is optimized for application in LED cooling by nanofluids, numerically. COMSOL-Multiphysics commercial code was used and Al_2_O_3_-water as the cooling nanofluid flow was selected as the working fluid. Three powers (1, 2 and 3 W) are used for the LED power output and to find the optimized arrangement of fins for reaching the minimum temperature (as cooling efficiency), Central composite design (CCD) was applied to propose minimum possible geometries (11 Cases). Finally, GFEM results of modeling were optimized by the Response Surface Method (RSM) which confirmed that the optimized case include parameters as Fin numbers = 8, Fin length = 20 and Fin thickness = 4 where nanoparticles with φ = 0.05 caused maximum cooling efficiency for this case by greater Nusselt number and lower pressure drop.

## References

[1] Ben Hamida MB, Charrada K (2012). Application of a three-dimensional model for a study of the energy transfer of a high-pressure mercury horizontal lamp. Phys. Plasmas..

[2] Ben Hamida MB, Helali H, Araoud Z, Charrada K (2011). Contrast between The vertical and horizontal mercury discharge lamps. Phys. Plasmas..

[3] Ben Hamida MB, Charrada K (2014). A three-dimensional thermal study of a mercury discharge lamp with double envelope for different orientations. J. Phys. Plasmas..

[4] Ferjani B, Ben Hamida MB (2019). Thermal study of the atomic ratio effect on a cylindrical and an ellipsoidal shaped HgTlI discharge lamps. Eur. Phys. J. D..

[5] Bensalah S, Ben Hamida MB (2019). Heat transfer enhancement of circular and square LED geometry. Int. J. Numer. Methods Heat Fluid Flow.

[6] Ben Salah S, Ben Hamida MB (2019). Alternate PCM with air cavities in LED heat sink for transient thermal management. Int. J. Numer. Methods Heat Fluid Flow.

[7] Ha M, Graham S (2012). Development of a thermal resistance model for chip-on-board packaging of high power LED arrays. Microelectron. Reliab..

[8] Ben Hamida MB, Charrada K, Almeshaal MA, Chamkha A (2021). A three-dimensional thermal analysis and optimization of square light edding diode subcomponents. Int. Commun. Heat Mass Transfer.

[9] Jia Y-L, Wang R, Zhang Y, Ma X-J, Fu-Xing Yu, Xiong Z-Y, Zhou D-Y, Xiong Z-H, Gao C-H (2019). Large current efficiency enhancement in the CsPbBr 3 perovskite light-emitting diodes assisted by an ultrathin buffer layer. J. Lumin..

[10] Fu-Xing Y, Yue Z, Zi-Yang X, Xing-Juan M, Ping C, Zu-Hong X, Chun-Hong G (2017). Full coverage all-inorganic cesium lead halide perovskite film for high-efficiency light-emitting diodes assisted by 1,3,5-tri(m-pyrid-3-yl-phenyl)benzene. Organ. Electron..

[11] Minqiang P, Haozhong H, Guanping D (2020). Experimental study of the performance of cutting copper fiber oriented sintered heat sinks for the water cooling of LEDs. Appl. Therm. Eng..

[12] Xiaohui L, Songping M, Bingzhong M, Lisi J, Ying C, Zhengdong C (2020). Thermal management of high-power LED based on thermoelectric cooler and nanofluid-cooled microchannel heat sink. Appl. Therm. Eng..

[13] Lin X, Mo S, Jia L, Yang Z, Chen Y, Cheng Z (2019). Experimental study and Taguchi analysis on LED cooling by thermoelectric cooler integrated with microchannel heat sink. Appl. Energy.

[14] Huang C-H, Wang G-J (2017). A design problem to estimate the optimal fin shape of LED lighting heat sinks. Int. J. Heat Mass Transf..

[15] Wang H, Jian Qu, Peng Y, Sun Q (2019). Heat transfer performance of a novel tubular oscillating heat pipe with sintered copper particles inside flat-plate evaporator and high-power LED heat sink application. Energy Convers. Manag..

[16] Tang Y, Lin L, Zhang S, Zeng J, Tang K, Chen G, Yuan W (2017). Thermal management of high-power LEDs based on integrated heat sink with vapor chamber. Energy Convers. Manag..

[17] Kim D, Lee J, Kim J, Choi C-H, Chung W (2015). Enhancement of heat dissipation of LED module with cupric-oxide composite coating on aluminum-alloy heat sink. Energy Convers. Manag..

[18] Park S-J, Jang D, Yook S-J, Lee K-S (2016). Optimization of a chimney design for cooling efficiency of a radial heat sink in a LED downlight. Energy Convers. Manag..

[19] Park S-J, Jang D, Lee K-S (2015). Thermal performance improvement of a radial heat sink with a hollow cylinder for LED downlight applications. Int. J. Heat Mass Transf..

[20] Junwei L, Ying Z, Debao Z, Shifei J, Zhuofen Z, Zhihua Z (2020). Model development and performance evaluation of thermoelectric generator with radiative cooling heat sink. Energy Convers. Manag..

[21] Ho CJ, Chung YN (2014). Chi-Ming Lai, Thermal performance of Al2O3/water nanofluid in a natural circulation loop with a mini-channel heat sink and heat source. Energy Convers. Manag..

[22] Cyril Reuben R, Suresh S, Vasudevan S, Chandrasekar M, Singh VK, Bhavsar RR (2020). Thermal performance of nano-enriched form-stable PCM implanted in a pin finned wall-less heat sink for thermal management application. Energy Convers. Manag..

[23] Hatami M, Ganji DD (2014). Thermal and flow analysis of microchannel heat sink (MCHS) cooled by Cu–water nanofluid using porous media approach and least square method. Energy Convers. Manag..

[24] Ben Hamida MB, Charrada K (2015). Natural convection heat transfer in an enclosure filled with an ethylene glycol-copper nanofluid under magnetic fields. Numer. Heat Transf. Part A Appl..

[25] Ben Jaballah R, Ben Hamida MB, Saleh J, Almeshaal MA (2019). Enhancement of the performance of bubble absorber using hybrid nanofluid as a cooled absorption system. Int. J. Numer. Meth. Heat Fluid Flow.

[26] Hatami M, Ganji DD (2013). Heat transfer and flow analysis for SA-TiO_2_ non-Newtonian nanofluid passing through the porous media between two coaxial cylinders. J. Mol. Liq..

[27] Zhou J, Hatami M, Song D, Jing D (2016). Design of microchannel heat sink with wavy channel and its time-efficient optimization with combined RSM and FVM methods. Int. J. Heat Mass Transfer.

[28] Tang W, Hatami M, Zhou J, Jing D (2017). Natural convection heat transfer in a nanofluid-filled cavity with double sinusoidal wavy walls of various phase deviations. Int. J. Heat Mass Transfer.

[29] Hatami M, Song D, Jing D (2016). Optimization of a circular-wavy cavity filled by nanofluid under the natural convection heat transfer condition. Int. J. Heat Mass Transf..

[30] Hatami M (2017). Nanoparticles migration around the heated cylinder during the RSM optimization of a wavy-wall enclosure. Adv. Powder Technol..

[31] Hatami M, Jing D (2017). Optimization of wavy direct absorber solar collector (WDASC) using Al 2 O 3-water nanofluid and RSM analysis. Appl. Therm. Eng..

[32] Hatami M, Zhou J, Geng J, Song D, Jing D (2017). Optimization of a lid-driven T-shaped porous cavity to improve the nanofluids mixed convection heat transfer. J. Mol. Liq..

[33] Massoudi MD, Ben Hamida MB, Almeshaal MA (2020). Free convection and thermal radiation of nanofluid inside nonagon inclined cavity containing a porous medium influenced by magnetic field with variable direction in the presence of uniform heat generation/absorption. Int. J. Numer. Meth. Heat Fluid Flow.

[34] Massoudi MD, Ben Hamida MB (2020). MHD natural convection and thermal radiation of diamond–water nanofluid around rotating elliptical baffle inside inclined trapezoidal cavity. Eur. Phys. J. Plus.

[35] Alsarraf J, Shahsavar A, Khaki M, Ranjbarzadeh R, Karimipour A, Afrand M (2020). Numerical investigation on the effect of four constant temperature pipes on natural cooling of electronic heat sink by nanofluids: A multifunctional optimization. Adv. Powder Technol..

[36] Shahsavar A, Ali G, Pouyan TS, Davood T, Hamzeh S (2019). Impact of variable fluid properties on forced convection of Fe3O4/CNT/water hybrid nanofluid in a double-pipe mini-channel heat exchanger. J. Therm. Anal. Calorim..

[37] Gheynani AR, Omid AA, Majid Z, Gholamreza ASS, Abdulwahab AA, Marjan G, Davood T (2019). Investigating the effect of nanoparticles diameter on turbulent flow and heat transfer properties of non-Newtonian carboxymethyl cellulose/CuO fluid in a microtube. Int. J. Numer. Methods Heat Fluid Flow.

[38] Kavusi H, Toghraie D (2017). A comprehensive study of the performance of a heat pipe by using of various nanofluids. Adv. Powder Technol..

[39] Gholami MR, Omid AA, Ali M, Davood T, Gholamreza ASS, Majid Z (2018). The effect of rib shape on the behavior of laminar flow of oil/MWCNT nanofluid in a rectangular microchannel. J. Therm. Anal. Calorim..

[40] Barnoon P, Davood T, Reza Balali D, Hossein A (2019). MHD mixed convection and entropy generation in a lid-driven cavity with rotating cylinders filled by a nanofluid using two phase mixture model. J. Magn. Magn. Mater..

[41] Toghraie D, Mahmoudi M, Akbari OA, Pourfattah F, Heydari M (2019). The effect of using water/CuO nanofluid and L-shaped porous ribs on the performance evaluation criterion of microchannels. J. Therm. Anal. Calorim..

[42] Arasteh H, Mashayekhi R, Toghraie D, Karimipour A, Bahiraei M, Rahbari A (2019). Optimal arrangements of a heat sink partially filled with multilayered porous media employing hybrid nanofluid. J. Therm. Anal. Calorim..

